# Association of Patients Reading Clinical Notes With Perception of Medication Adherence Among Persons With Serious Mental Illness

**DOI:** 10.1001/jamanetworkopen.2021.2823

**Published:** 2021-03-24

**Authors:** Charlotte Blease, Zhiyong Dong, John Torous, Jan Walker, Maria Hägglund, Catherine M. DesRoches

**Affiliations:** 1 Division of General Medicine, Beth Israel Deaconess Medical Center, Boston, Massachusetts; 2 Harvard Medical School, Boston, Massachusetts; 3 Department of Psychiatry, Beth Israel Deaconess Medical Center, Boston, Massachusetts; 4 Department of Women’s and Children’s Health, Uppsala University, Uppsala, Sweden

## Abstract

This survey study examines how patients with a mental illness diagnosis who read at least 1 clinical note in the last year perceived its association with their medication adherence.

## Introduction

Starting April 5, 2021, new US federal rules will mandate that all clinicians (including physicians, physician assistants, and nurse practitioners) must share clinical notes with patients via online health portals with few exceptions.^1^ Surveys show that clinicians worry that patients with mental health diagnoses will become anxious, confused, or upset after reading their visit notes.^2^ In this study, we examined how patients with a mental illness diagnosis who read at least 1 clinical note in the last 12 months perceived how reading the note affected their adherence to prescribed medication.

## Methods

We reanalyzed data from a web-based survey of patient experiences with access to their outpatient visit notes.^3^ Data were collected in 2017 from adult patients in 3 diverse health systems in the US, who had accessed at least 1 ambulatory note in the previous 12 months. All participants gave written informed consent. The institutional review boards at Beth Israel Deaconess Medical Center in Boston, Massachusetts, Geisinger Health System in Pennsylvania, and the University of Washington Medicine in Seattle approved the survey and study protocol at their respective sites. The American Association for Public Opinion Research (AAPOR) reporting guideline was used in this survey study.

Of the 136 815 patients who received a survey invitation, 29 656 responded for a response rate of 22% using the AAPOR guidelines.^4^ We excluded all participants who did not report taking or being prescribed medication in the 12 months before the survey. While there is no consensus or federal definition of serious mental illness (SMI) in the US, the term is often used to describe major depression, bipolar, and schizophrenia-related disorders.^5^ Using the *International Statistical Classification of Diseases and Related Health Problems, Tenth Revision *(*ICD-10*), we classified each respondent as having (1) SMI (major depressive disorder, schizophrenia, schizoaffective disorder, or bipolar-related disorder [*ICD-10* codes F20.0-F29 and 31.0-F33.9]), (2) other mental illness (*ICD-10 *codes F00-F99, except for those listed earlier), or (3) no mental illness. We used a 2-sided *t* test, χ^2^ test, and Fisher exact test for statistical analyses, and significance was set at *P* < .001. All analyses were completed using SAS software version 9.4 (SAS Institute) and performed between August 2020 and January 2021.

## Results

In the sample of 29 656 respondents, 23 576 (79%) had read at least 1 note, and among them, 19 411 (82%) were taking or had been prescribed a medication in the past 12 months. Diagnostic codes were missing for 31 participants, leaving an analytic sample of 19 380; among the respondents 12 112 (62%) were women and 14 775 (76%) were aged 45 years or older. Of 18 943 patients with known race/ethnicity, 16 132 (85%) were White patients, 961 (5%) were Asian patients, 701 (4%) were Hispanic patients, and 496 (3%) were Black patients. Of the full sample of 19 380, 1371 (7%) had a diagnosis of SMI, and 1742 (9%) had another mental illness. Compared with patients without a mental health diagnosis, those with an SMI diagnosis and those with other mental health diagnoses were more likely to have been prescribed or take medications in the past 12 months (16 267 of 18 731 [87%] vs 1371 of 1415 [97%] vs 1742 of 1856 [94%], respectively; *P* < .001). Patients with SMI viewed their notes at a rate of 47% (95% CI, 46%-49%) compared with patients without a mental health diagnosis, who viewed notes at a rate of 62% (95% CI, 62%-63%) (*P* < .001) (Table 1). Among participants with an SMI diagnosis, 84 of 427 (20%) reported that reading their notes made them more likely to take their medications compared with 1317 of 9670 (14%) of patients with no mental health diagnosis (*P* < .001) (Table 2). Among patients with an SMI diagnosis, 898 of 1331 respondents (67%) reported that reading notes helped them understand why medications were prescribed; 882 of 1313 respondents (67%) felt more in control of their medications; 856 of 1319 respondents (65%) reported feeling more comfortable with their medications; and 789 of 1322 respondents (60%) reported that their notes helped answer their medication questions. Few respondents (23 of 1308 [2%] of those with SMI to 45 of 1653 [3%] of those with other mental health diagnosis) reported that reading their notes made them more confused; however, patients with an SMI diagnosis (61 of 1314 [5%]) and those with other mental health diagnoses (85 of 1646 [5%]) were more likely to report feeling more worried compared with patients without mental health diagnoses (524 of 15 233 [3%]; *P* < .001).

**Table 1.** Rate of Reading Visit Notes

Outcome measures	SMI diagnosis (n = 1371)	Other MH diagnosis (n = 1742)	No MH diagnosis (n = 16 267)
Notes per patient, mean (SD), No.	32.23 (49.17)	21.54 (24.55)	13.76 (18.53)
Notes viewed per patient, mean (SD), No.	16.38 (41.74)	11.32 (16.41)	7.75 (12.63)
Proportion of available notes viewed			
Mean (SD [95% CI])	0.47 (0.29 [46-49])	0.55 (0.30 [53-56])	0.62 (0.30 [62-63])
*P* value^a^	<.001	<.001	NA

Abbreviations: MH, mental health; NA, not applicable; SMI, serious mental health illness.

^a^ The* t* test was used to compare the mean difference of note viewing rate between the SMI group vs no MH group as well as the other MH group vs no MH group.

**Table 2.** Patients’ Self-reported Experiences of Reading Visit Notes and Managing Medications

Perception	Patients, No. (%)			*P* value	
	SMI diagnosis (n = 1371)	Other mental health diagnosis (n = 1742)	No mental health diagnosis (n = 16 267)	Across all groups	SMI vs no mental health diagnosis
**Positive statements**					
More likely to take my medications as prescribed, No./total No.(%)^a^	84/1532 (20)	131/1532 (18)	1317/1532 (14)	<.001	.003^b^
Helped me understand why medications were prescribed	898 (67)	1106 (66)	9987 (64)	.01	.01
Made me more comfortable with my medications	856 (65)	1040 (63)	9296 (61)	<.001	.002
Made me seek more information about my medications	514 (39)	570 (34)	4808 (31)	<.001	<.001^c^
Answered my questions about medications	789 (60)	963 (58)	8679 (57)	.05	.03
Made me feel more in control of my medications	882 (67)	1077 (65)	9275 (61)	<.001	<.001
Helped me understand possible side effects of my medications	662 (50)	775 (47)	6841 (45)	<.001	<.001
**Negative statements**					
Made me confused about my medications	23 (2)	45 (3)	266 (2)	.02	.95
Made me worried about my medications	61 (5)	85 (5)	524 (3)	<.001	.02

^a^ This question was only asked at 2 of 3 health systems (Beth Israel Deaconess Medical Center, Boston, and Geisinger Health System, Pennsylvania); therefore, the proportion was calculated using the denominator from only these health systems. All other questions were asked at all 3 health systems, which included the University of Washington Medicine, Seattle.

^b^ *P* value for this figure was calculated with Fisher exact test because of the reduced sample size for cell frequencies, which were not indicated in Table 2 of less than 5. Two of the patients with severe mental health conditions responded that they were less likely to take medications as prescribed, and this category was not included in the Table. All other *P* values in the Table were calculated using χ^2^ statistic.

^c^ The level of significance was set at <.001 because of the study’s large sample size.

## Discussion

Among patients with SMI, access to clinicians’ notes may help clarify why medications have been prescribed and improve understanding of possible side effects. The findings of this study are promising in light of evidence that rates of psychotropic medication adherence for patients with major depressive disorders, bipolar disorders, and schizophrenia are only approximately 50%.^6^

This study has several limitations. The survey did not determine the kinds of medication on which participants based their responses. While the definition we used for SMI was supported by the literature and the Substance Abuse and Mental Health Administration, the results may vary based on the definition. Because the survey was reliant on self-report measures, it was unknown whether response biases affected the survey. While the response rate was moderate for an online survey, the demographic breakdown and sampling restriction to 3 US health systems were further limitations.

Sharing mental health notes will present challenges, and understandably, clinicians express concerns about patient access to their notes. However, access to clinical notes may benefit patients’ understanding and knowledge about their medications, including among persons with SMI.
